# *ATAD3B* and *SKIL* polymorphisms associated with antipsychotic-induced QTc interval change in patients with schizophrenia: a genome-wide association study

**DOI:** 10.1038/s41398-022-01825-0

**Published:** 2022-02-08

**Authors:** Zhe Lu, Yuyanan Zhang, Hao Yan, Yi Su, Liangkun Guo, Yundan Liao, Tianlan Lu, Hao Yu, Lifang Wang, Jun Li, Wenqiang Li, Yongfeng Yang, Xiao Xiao, Luxian Lv, Yunlong Tan, Dai Zhang, Weihua Yue

**Affiliations:** 1 Institute of Mental Health, Peking University Sixth Hospital, Beijing, 100191 China; 2 National Clinical Research Center for Mental Disorders (Peking University Sixth Hospital), Beijing, 100191 China; 3 NHC Key Laboratory of Mental Health, & Research Unit of Diagnosis and Treatment of Mood Cognitive Disorder (2018RU006), Chinese Academy of Medical Sciences, Beijing, 100191 China; 4 Peking University HuiLongGuan Clinical Medical School, Beijing HuiLongGuan Hospital, Beijing, 100096 China; 5 Department of Psychiatry, Jining Medical University, Jining, Shandong 272067 China; 6 Henan Key Lab of Biological Psychiatry, The Second Affiliated Hospital of Xinxiang Medical University, Xinxiang, Henan 435001 China; 7 Key Laboratory of Animal Models and Human Disease Mechanisms of the Chinese Academy of Sciences and Yunnan Province, Kunming Institute of Zoology, Chinese Academy of Sciences, Kunming, Yunnan 650223 China; 8 PKU-IDG/McGovern Institute for Brain Research, Peking University, Beijing, 100871 China; 9 Chinese Institute for Brain Research, Beijing, 102206 China

**Keywords:** Clinical genetics, Schizophrenia

## Abstract

QTc interval prolongation is one of the most common antipsychotic-induced side effects which could lead to ventricular tachycardia or Torsade de Pointes, even cardiac arrest. There is very limited understanding on the genetic factors that associated with antipsychotic-induced QTc interval change. We conducted a genome-wide association study (GWAS) of antipsychotic-induced QTc interval change among patients with schizophrenia. A total of 2040 patients with schizophrenia were randomly assigned to six groups (olanzapine, risperidone, quetiapine, aripiprazole, ziprasidone, and first-generation antipsychotics; first-generation antipsychotics including haloperidol or perphenazine were also assigned randomly) and received 6-week antipsychotic treatment. We identified two novel loci (rs200050752 in *ATAD3B* and rs186507741 in *SKIL*) that were associated with antipsychotic-induced QTc interval change at a genome-wide significance level. The combination of polygenic risk score (PRS), based the GWAS of myocardial infarction from BioBank Japan project, and clinical data (sex, heart rate and QTc interval at baseline) could be applied to predict whether patients with schizophrenia have QTc interval prolongation (10 ms was applied as threshold, *P* < 0.001, area under the curve [AUC] was 0.797), especially for the first episode patients (*P* < 0.001, AUC was 0.872). We identified two loci located within genes related to mitochondrial function and cell growth and differentiation, which were both associated with schizophrenia and heart function. The combination of PRS and clinical data could predict whether patients with schizophrenia have the side effect of QTc interval prolongation, which could fundamentally guide the choice of antipsychotic in patients with schizophrenia, especially for the first-episode patients.

## Introduction

Schizophrenia is a severe mental disorder, with the lifetime prevalence of around 1% worldwide, which is characterized by a range of perception, sensation, cognitive, behavioral, and emotional dysfunction [1]. Antipsychotics are the first-line therapy in the clinical management of schizophrenia. While many patients benefited from the antipsychotics, approximate 75% of patients discontinue treatment due to lack of efficacy, low compliance or side effects, which prolongs the optimum treatment time and influences the prognosis [2, 3].

Persons with schizophrenia are at a higher risk of mortality rate than the general population [4]. Sudden cardiac death is a significant concern in patients with schizophrenia, which can occur due to cardiac arrhythmias like Torsade de Pointes (TdP) resulting from an increased cardiac repolarization time. The QTc interval and T wave abnormalities reflect increased repolarisation duration [5]. Some kinds of antipsychotics could induce the QTc prolongation, which might lead to ventricular tachycardia or TdP [6]. Furthermore, most guidelines apply the QTc interval prolongation as an indicator to predict the risk of antipsychotic-induced serious cardiac arrhythmias. Several antipsychotics were restricted in the clinical management because of the QTc prolongation. For example, amisulpride, ziprasidone and quetiapine seem to prolong the QTc interval more than other antipsychotics [7].

Evidence suggested that genetic factors played a consequential role in the QTc interval [8]. A meta-analysis of genome-wide association study (GWAS) in 76,061 European ancestry individuals were conducted by the QT Interval-International GWAS Consortium identified 35 loci related to QT interval, which highlighted the importance of calcium regulation in myocardial repolarization [9]. The recent transethnic GWAS on long QT syndrome identified three genome-wide significant loci near *NOS1AP* (rs12143842), *KCNQ1* (rs179405), and *KLF12* (rs17061696) [10]. However, little was known about genetic factors on antipsychotic-induced QTc interval prolongation at the genome-wide level. Previous GWAS of iloperidone-induced QTc interval prolongation identified 6 loci, but these loci were not at genome-wide statistical significance [11]. Another five-antipsychotic-specific GWAS including 738 patients with schizophrenia identified one locus near *SLC22A23* (rs4959235) associated with quetiapine-induced QTc prolongation, while it neither at genome-wide statistical significance [12].

The results of previous studies on antipsychotic-induced QTc interval change were unsatisfactory, which may result from the small sample size. Besides, there was no similar study on the Chinese Han population. To improve the understanding of genetic factors on antipsychotic-induced QTc change, we conducted the GWAS of antipsychotic-induced QTc interval change, including 2040 participants, in a 6-week randomized controlled clinical trial evaluating the efficacy, safety, and tolerability of seven antipsychotics (included olanzapine, risperidone, quetiapine, aripiprazole, ziprasidone, haloperidol, and perphenazine), in patients with schizophrenia.

## Methods

### Subjects

The cohort was from the Chinese Antipsychotics Pharmacogenomics Consortium (CAPOC), including five research centers, leading a total of 32 psychiatric hospitals in China. This study was compliant with the Declaration of Helsinki. The protocol was approved by the Clinical Research Ethics Committee at each site, and written informed consent was obtained.

### Inclusion criteria

(1) Diagnosed with schizophrenia based on the Structured Clinical Interview of the Diagnostic and Statistical Manual of Mental Disorders, fourth edition (DSM-IV); (2) Aged from 18 to 45 years; (3) Han Chinese ancestry; (4) Total scores of the Positive and Negative Syndrome Scale (PANSS) were more than 60, and three positive items scored more than four at least; (5) Physically healthy with all laboratory parameters within normal limits; (6) Could be treated with oral medication; (7) Provide informed consent.

### Exclusion criteria

(1) Diagnosed with other mental disorders met the criteria of DSM-IV; (2) With unstable physical diseases, malignant syndrome or acute dystonia, well-documented histories of epilepsy and hyperpyretic convulsion; (3) Required long-acting injectable antipsychotics; (4) Regularly toke with clozapine during the past month; (5) Treated with electroconvulsive therapy during the last month; (6) Had previously attempted suicide, or had experienced the symptoms of severe excitement and agitation; (7) Abnormal liver or renal function; (8) without legal guardian; (9) Had QTc prolongation, a history of congenital QTc prolongation, or myocardial infarction within the past 6 months; (10) Pregnant or breastfeeding; (11) Had a contraindication to any of the drugs to which they could be assigned.

### Randomization

Eligible patients were randomly assigned (1:1:1:1:1:1) to six groups (aripiprazole, olanzapine, quetiapine, risperidone, ziprasidone, or one of the first-generation antipsychotics [haloperidol or perphenazine]). Those randomly assigned to the first-generation antipsychotics group were subsequently randomly assigned (1:1) to haloperidol or perphenazine.

### Clinical Procedure

Within 2 weeks of randomization, psychiatrists from the study adjusted the dosages on the basis of treatment effectiveness, in keeping with the study protocol. The dosage of antipsychotics then remained unchanged throughout the study. If the psychiatrists decided that a patient’s response was not adequate or the patient decided to drop out of the study, treatment was discontinued and the last observation was carried forward to represent treatment response. Patients with adequate responses continued treatment until the end of the study.

### Genotyping

The samples were genotyped with Illumina Human Omni ZhongHua-8 Beadchips (Illumina, San Diego, CA, USA), which were designed for Chinese populations. Quality control was done before the association analysis. Genotype imputation for the sample was done with the pre-phasing imputation stepwise approach implemented in IMPUTE2 and SHAPEIT (Version 2.r727). Haplotypes derived from phase I of the 1000 Genomes Project (release version 3) were used as references.

Detailed randomization, masking, clinical and genotyping procedures had been described in the previous CAPOC study [13] and enclosed in the appendix.

### QTc interval

Twelve-lead electrocardiography was measured at baseline and the end of the 6^th^ week. The QT interval needs to be corrected for heart rate to make it more clinically meaningful. Bazett’s formula is the most common method used to correct QT interval for heart rate. However, the Bazett’s formula overestimates QTc interval at heart rates higher than 60 and underestimates at rates lower than 60. The International Council for Harmonization of Technical Requirements for Pharmaceuticals for Human Use (ICH) recommended using Fridericia’s formula which is less influenced by extremes of heart rate to correct the QT interval. In our study, QTc interval was calculated based on Fridericia’s formula [14].

### Statistical analysis

We did the linear regression in PLINK (version 1.9) to assess the associations between allele and QTc interval change [15]. Sex, age, research centers, type of antipsychotics, the dosage of antipsychotics (chlorpromazine equivalent dose equivalence [16]), course of schizophrenia at baseline and the first five principal components of population structure (PCA) were set as covariates. Briefly, we hypothesized that the effect of different antipsychotics on QTc interval change was on the general pathways, and assessed the associations between genotype and QTc interval change across the whole sample. Then we examined the antipsychotic-specific effect on QTc interval change. The accepted genome-wide significance threshold of a *P* value of less than 5 × 10^−8^ were adopted [17], and the associations with a *P* less than 1 × 10^−5^ were set as findings of interest.

Furthermore, multiple several secondary analyses were carried out on the genome-wide association results. Firstly, the expression patterns of certain genes in human tissues were explored, mainly focus on brain, heart and artery tissues, with the Genotype-Tissue Expression database [18]. The protein expression pattern was searched in the Human Protein Atlas (https://www.proteinatlas.org/). And we searched the eQTL effect of the identified single-nucleotide polymorphisms (SNPs) in three database including GTExPortal (https://www.gtexportal.org/), BRAINEAC (http://www.braineac.org/) and Blood eQTL browser (https://www.genenetwork.nl/bloodeqtlbrowser/).Then we used the suggested SNPs to conduct the pathways and diseases enrichment analysis on the Metascape website (http://metascape.org/).[19]

Finally, the polygenic risk scores (PRS) were calculated based on the SNP effect sizes of the myocardial infarction (MI) from the GWAS included 1,666 individuals with MI and 3,198 referents from BioBank Japan project [20], and we combined the PRS (Sex, age, research centers, type of antipsychotics, the dosage of antipsychotics, course of schizophrenia, and the first five principal components of PCA were defined as covariates) and some clinical data including sex, heart rate, QTc interval at baseline to distinguish whether patients with schizophrenia have the QTc interval prolongation after antipsychotics treatment. Prolongation more than 10 ms after 6-week antipsychotic treatment was as the threshold.

## Results

**Fig. 1:**
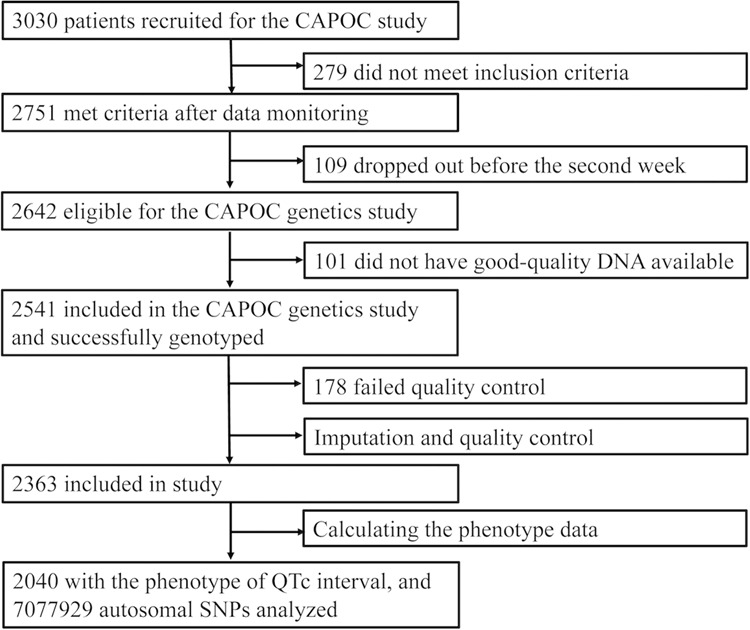
Trial profile for the cohort. CAPOC Chinese Antipsychotics Pharmacogenomics Consortium, SNP single-nucleotide polymorphism.

Total 2040 patients had the complete data of QTc interval change and passed the quality control. The flow chart of the study was described by the Fig. 1, and demographic and clinical characteristics were displayed in the Table 1. The difference of sex, age and CHL-equivalent dose among seven groups were significant, while there were no significant differences in rate of first-episode, course of schizophrenia and baseline QTc interval. Supplementary Figure 1 showed the Manhattan and quantile–quantile plots of the whole sample. Total 68 SNPs were modest associated with change of QTc interval (*P* < 1 × 10^−5^). Part of 68 SNPs did in linkage disequilibrium, and the 68 SNPs were clumped in PLINK with a cutoff *r*² of 0.2 within a 250-kb window, 35 clumps formed from 68 top variants (We showed the LD block plot in Supplementary Fig. 2).

**Table 1:** Demographic and clinical characteristics (Data are *n* (%) or mean (SD)).

	Risperidone	Olanzapine	Quetiapine	Aripiprazole	Ziprasidone	Perphenazine	Haloperidol	*F/χ*^*2*^	*P*
	*n* = 354	*n* = 356	*n* = 333	*n* = 340	*n* = 339	*n* = 170	*n* = 148		
Age (years)	30.85 (7.95)	30.23 (7.90)	30.89 (7.76)	31.31 (7.66)	30.48 (7.86)	32.07 (7.83)	33.47 (7.46)	3.926	0.001
Sex								15.493	0.017
Male	212 (59.9%)	183 (51.4%)	155 (46.5%)	168 (49.4%)	173 (51.0%)	81 (47.6)	71 (48.0%)		
Female	142 (40.1%)	173 (48.6%)	178 (53.5%)	172 (50.6%)	166 (49.0%)	89 (52.4%)	77 (52.0%)		
First episode	102 (28.8%)	95 (26.7%)	82 (24.6%)	96 (28.2%)	90 (26.5%)	39 (22.9%)	33 (22.3%)	4.541	0.604
CHL-equivalent dose at Endpoint (mg/day)	270.68 (103.85)	506.88 (205.50)	442.57 (165.99)	429.41 (249.52)	387.83 (200.44)	253.59 (132.67)	503.97 (274.11)	71.501	<0.001
Course of schizophrenia (month)	75.48 (68.14)	78.38 (70.76)	83.09 (73.57)	76.86 (68.56)	73.03 (67.29)	90.14 (76.82)	89.61 (68.76)	2.070	0.054
Baseline QTc (ms)	405.24 (25.92)	403.71 (23.55)	406.63 (27.51)	405.11 (25.12)	404.07 (25.09)	405.82 (25.91)	404.96 (27.93)	0.482	0.822

*CHL* chlorpromazine.

There were two SNPs showed genome-wide significant associations with change of QTc interval (*P* < 5 × 10^−8^), in *ATAD3B* (rs200050752, missense) and *SKIL* (rs186507741, intronic) (Supplementary Fig. 3, Table 2). The QTc interval change was significantly different across seven antipsychotics (*F* = 7.086, *P* < 0.001). In the post hoc analyses (*Bonferroni test*), patients given quetiapine or ziprasidone had the most QTc interval prolongation (Table 3). QTc interval change of quetiapine and ziprasidone were more than aripiprazole and risperidone, and the change of QTc interval in the olanzapine subgroup was more than aripiprazole subgroup. The *post-hoc* test showed that the difference of QTc interval change between the two kinds of first-generation antipsychotics and the differences between the five second-generation antipsychotics and the two first-generation antipsychotics were not significant.

**Table 2:** Genome-wide association results of QTc interval change.

CHR	SNP	Position	Minor allele	Major allele	MAF	Functional annotation	Nearby gene	*β*	SE	*P* value
1	rs200050752	1414070	T	C	0.01	Missense	*ATAD3B*	36.02	5.535	3.559 × 10^−8^
3	rs186507741	170088226	C	A	0.01	Intronic	*SKIL*	24.03	5.477	4.909 × 10^−8^

*CHR* Chromosome, *SNP* single-nucleotide polymorphism, *MAF* frequency of minor allele.

**Table 3:** Outcomes of QTc change across seven antipsychotic drugs.

Measurement	Risperidone	Olanzapine ^a^	Quetiapine ^a^	Aripiprazole ^a^	Ziprasidone ^a^	Perphenazine	Haloperidol	*F*	P*
QTc change (ms)	*n* = 354	*n* = 356	*n* = 333	*n* = 340	*n* = 339	*n* = 170	*n* = 148	7.086	<0.001
Mean (SD)	−2.39 (26.27)	3.47 (28.61)	6.17 (33.35)	−6.08 (30.95)	4.85 (32.03)	−0.33 (32.22)	−1.68 (25.22)		
Post hoc test *(Bonferroni test)*	Risperidone < Quetiapine (*P* = 0.004) and Ziprasidone (*P* = 0.033); Olanzapine > Aripiprazole (*P* = 0.001); Quetiapine > Risperidone (*P* = 0.004) and Aripiprazole (*P* < 0.001); Aripiprazole < Olanzapine (*P* = 0.001), Quetiapine (*P* < 0.001) and Ziprasidone (*P* < 0.001); Ziprasidone > Risperidone (*P* = 0.033) and Aripiprazole (*P* < 0.001). The post-hoc test showed that the difference of QTc interval change between the two kinds of first-generation antipsychotics and the differences between the five second-generation antipsychotics and the two first-generation antipsychotics were not significant.								

^*^ The overall *P* value is for the comparison of seven groups with the use of ANOVA test. If the difference among the groups was significant at a *P* value of less than 0.05, the seven groups were compared with each other to identify significant differences (*P* < 0.05) between groups.

^a^ referred to the *P* value of *paired t test* after treatment in seven subgroup was significant.

The two genes with genome-wide significance were both remarkably expressed in brain tissues, heart tissues and artery tissues (Supplementary Fig. 4). The protein expression of *ATAD3B* gene was at medium level in the brain and cardiomyocytes, while the protein expression of *SKIL* gene was at low level in the brain and was not detected in cardiomyocytes. We tried to search the eQTL effect of the two SNPs based on the GTExPortal, BRAINEAC and Blood eQTL browser, however, both the two SNPs were not associated with gene expression.

In the pathway analysis, 68 SNPs were annotated to 20 genes, and then the 20 genes were enriched in two pathways: positive regulation of cell development and spermatogenesis (Supplementary Table 1). In the enrichment analysis in DisGeNET (http://www.disgenet.org), the 20 genes were enriched in seven diseases or clinical traits: creatinine measurement, glomerular filtration rate, malignant head and neck neoplasm, cardiac arrest, scoliosis, and mood disorders (Supplementary Table 1).

**Fig. 2:**
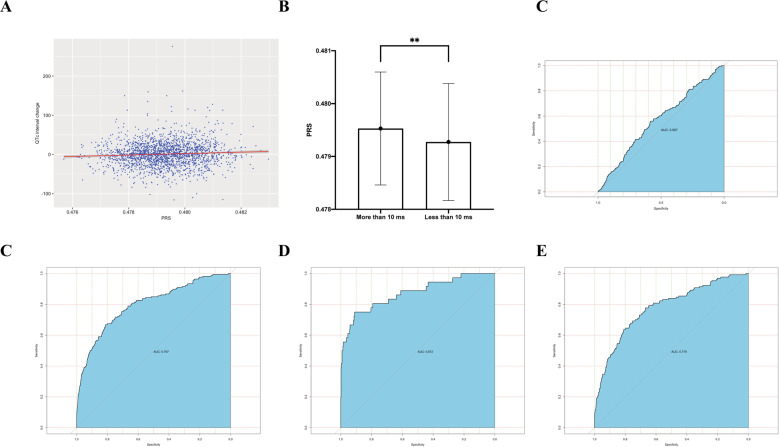
Predictive effect of the PRS and clinical data. **A** The association between QTc interval change and PRS was positive (*r* = 0.067, *P* = 0.003). **B** Patients with QTc interval prolongation (applied 10 ms as threshold) had the higher level of PRS (*t* = 2.865, *P* = 0.004). **C** ROC analysis indicated that the PRS could significantly predict whether patients with schizophrenia have QTc prolongation after antipsychotic treatment (threshold: 10 ms). **D** The combination of PRS and clinical data could improve the effect of prediction. **E** and **F** were the ROC analysis for the first-episode subgroup and relapse subgroup separately.

We calculated the PRS based on the GWAS of MI from BioBank Japan project, the threshold was 0.4957, included 55623 SNPs (*P* = 0.021, PRS_*R*^2^ = 0.003) (Supplementary Fig. 5). The positive association between QTc interval change after 6-week-antipsychotic treatment and the best PRS was significant (*r* = 0.067, *P* = 0.003) (Fig. 2A). Moreover, patients with QTc interval prolongation (applied 10 ms as threshold [21, 22]) had the higher level of PRS (*t* = 2.865, *P* = 0.004) (Fig. 2B). We applied the PRS to predict whether patients with schizophrenia have the QTc interval prolongation after antipsychotic treatment (applied 10 ms as threshold), the area under the curve (AUC) was 0.567 and the *P* value was 0.004 (Fig. 2C). Then we performed the *Path analysis* to detect some clinical indicators which could affect the QTc interval change, including sex, age, course of schizophrenia, blood pressure, heart rate, weight, body mass index, abdominal circumference, PR interval, QRS interval and QTc interval at baseline. The result showed that only the sex, heart rate and QTc interval at baseline had a significant direct effect on antipsychotic-induced QTc interval change. Furthermore, there were indirect effect between sex and baseline QTc interval, as well as baseline QTc interval and baseline heart rate (Supplementary Fig. 6). And then we did a *Logistic analysis* using PRS and the clinical data (sex, heart rate and QTc interval at baseline) to distinguish whether patients with schizophrenia have the QTc interval prolongation after antipsychotic treatment. The result showed that the combination of PRS, sex, heart rate and QTc interval at baseline could significantly distinguish whether patients with schizophrenia have the QTc prolongation at the threshold of 10 ms (*P* < 0.001, AUC was 0.797, Fig. 2D). When we conducted stratified Receiver Operating Characteristic (ROC) analysis by patients were first episode or not, the result indicated that the distinguish ability of PRS and clinical data was more powerful at first-episode subgroup (AUC of the first-episode subgroup: 0.872; AUC of relapse subgroup: 0.778) (Fig. 2E, F).

## Discussion

In our study, we found the QTc interval increased in the olanzapine, quetiapine and ziprasidone subgroups after 6-week treatment, while it decreased in the aripiprazole subgroups, the QTc interval change was not significant in risperidone, perphenazine and haloperidol subgroups. Moreover, the QTc change of quetiapine and ziprasidone were more than aripiprazole and risperidone subgroups. The results in olanzapine, quetiapine, ziprasidone and aripiprazole were comparable to the meta-analysis of 32 antipsychotics [7], while the QTc interval change in the risperidone and haloperidol subgroups were inconsistent. The inconsistent result might result from the short follow-up duration. The antipsychotics, which have a larger effect on QTc interval, have shown the significant change after the 6-week treatment such as ziprasidone.

Based on the literature, this study is so far the largest genome-wide association study of antipsychotic-induced QTc interval change. We identified two loci associated with antipsychotic-induced QTc interval change, which located within genes related to mitochondrion functions and regulation of cell growth and differentiation.

The missense SNP rs200050752 is located within the ATPase Family AAA Domain Containing 3B (*ATAD3B*) gene. The protein, encoded by *ATAD3B* localizes to the mitochondrial inner membrane, may involve in the mitochondrial function, which was characterized by reduced mitochondrial metabolism, low mitochondria DNA (mtDNA) copies and fragmented mitochondrial network. The mtDNA encodes a series of critical subunits of the oxidative phosphorylation as well as tRNAs and rRNAs essential to their synthesis, and plays a substantial role in innate immune responses and inflammatory pathology [23, 24]. Each cell carries hundreds of mtDNA copies, which is packaged by a series of proteins, including TFAM, POLG, prohibitins, and ATAD3, to form an mtDNA-protein complex called the nucleoid [25]. Damage of the mtDNA occurs in response to a series of physiological stresses, so the mtDNA is susceptible to damage by oxygen reactive species [26]. These mechanisms contribute to the very high mutation rate of the mtDNA, which is about 10- to 17-fold higher than that in the nuclear DNA [27, 28]. The mtDNA mutations and mitochondrial dysfunction are associated with various human diseases, ranging from severe inherited disorders to common late-onset diseases, such as mitochondrial encephalopathy [29]. The heart is mainly composed of cardiomyocytes containing a large quantity of mitochondria [30], an impaired energy balance from mitochondria can be generally recognized as both a cause and an effect of heart dysfunction [31, 32]. Recent studies have increased the understanding of mitochondrial genetics, providing an insight into the correlations between mitochondrial mutations and cardiac manifestations including hypertrophic or dilated cardiomyopathy, arrhythmia (including QTc interval prolongation), autonomic nervous system dysfunction, heart failure, or sudden cardiac death [33]. The *ATAD3* gene family in humans positioned on chromosome 1p36.33 and includes three paralogs (*ATAD3A, ATAD3B* and *ATAD3C*). *ATAD3* cluster deletions could lead to cerebellar dysfunction associated with altered mtDNA and cholesterol metabolism [34] whose clinical manifestation is the phenotypic spectrum covering developmental delay, hypotonia, optic atrophy, peripheral neuropathy, and hypertrophic cardiomyopathy, epilepsy. Brain energy metabolism, mitochondrial functions, and redox balance are also associated with psychiatric disorders. It is possible that they are implicated in the etiology and progression of psychiatric disorders [35, 36]. Moreover, we also searched allele frequencies of the SNP rs200050752 with reference to the 1000 Genomes Project Phase 3, and found that the allele frequencies in East Asian populations were 4%, while it was zero in other populations. The participants in previous studies were all European ancestry, so it is necessary to conduct the GWAS in the Chinese population.

The intronic SNP rs186507741 is located within SKI like proto-oncogene (*SKIL*). The protein encoded by *SKIL* is a component of the SMAD pathway, playing a role in the regulation of cell growth and differentiation through transforming growth factor-beta (TGF-β) [37]. The cytokine TGF-β mediates tissue fibrosis associated with inflammation and tissue injury, fibroblast-specific TGF-β-SMAD2/3 signaling underlies cardiac fibrosis [38]. Moreover, persons with TGFβR2 mutations carried a high prevalence of ventricular repolarization abnormalities [39], and the TGF-β inhibitor therapy could decrease fibrosis and stimulates cardiac improvement [40]. A recent RNA sequencing study identified two microRNAs (miR) involved in infarction (MI), *miR-30-5p* downregulated and *miR-142a-5p* upregulated respectively in MI, and the *vitro* experiment demonstrated that *miR-30-5p* is anti-apoptotic and *miR-142a-5p* is pro-apoptotic; then their luciferase assays displayed that the *Picalm* and *Skil* (apoptotic genes), and the *Ghr* and *Kitl* (anti-apoptotic genes) are direct targets of *miR-30-5p* and *miR-142a-5p*, respectively [41]. Another study demonstrated that, both in vitro and in vivo, inhibiting miR-155 expression and Ski/SnoN signaling could lead to aggravated cardiac fibrogenesis response after MI, which was through the TGF-β pathway [42]. As for the association between the *SKIL* and schizophrenia, a previous study used the network coexpression analysis in three microarray datasets, and established a miRNA-TF-gene network related to schizophrenia, including the *EGR1*-miR-124-3p-*SKIL* feed-forward loop. Their real-time quantitative PCR analysis illuminated patients with schizophrenia have the overexpression of miR-124-3p, the under expression of *SKIL* and *EGR1* in the blood compared with controls, and after a 12-week treatment, the direction of change of miR-124-3p and *SKIL* mRNA levels were reversed [43].

The antipsychotic-specific analyses suggested that *GRM1* and *USP44* was associated with QTc change to risperidone, *KDM2B*, *LINC00469* and *EBF2* with QTc change to olanzapine, *ADK* and *OTOGL* with QTc change to quetiapine, *CACNA1C, URGCP, SNORA2B, KANSL2, BLVRA, PPM1H, DAB1, ASB2*, and *KAT2B* with QTc change to aripiprazole, *CRK, LINC01237, LINC00907, RALYL*, and *GAB1* with QTc change to ziprasidone, while there were no genome-wide significant SNPs associated with QTc change to perphenazine and haloperidol (Supplementary Table 2). And we conducted the pathway and disease enrichment by the Metascape, these genes could be enriched in the pathway of actin filament-based process (GO: 0030029) and heart development (GO: 0007507); moreover, these genes could be enriched in the diseases of viral myocarditis (C0276138) and cardiac arrest (C0018790). Furthermore, genes (including *GRM1, ADK, CACNA1C, CRK* and *GAB1*) which the most significant SNPs of the five first-generation antipsychotics specific analyses are located within were both associated with the schizophrenia and heart diseases [44–52]. For instance, *CACNA1C* is an important gene for schizophrenia, encoding the L-type calcium channel Cav1.2, which is a target of available schizophrenia drugs, as well as the heart repolarization function. However, there was no overlap among identified genome-wide loci affecting the specific effect on the QTc interval for each of the five drugs. There were two probable possibilities. First, antipsychotic drugs may work via common pathways, which can be mediated by only a small number of genes. Second, different antipsychotics act via different mechanisms, for example, olanzapine is a broad-spectrum receptor antagonist, risperidone is a dopamine receptor D2 (DRD2) and serotonin (5HT) 2 receptor antagonist, while aripiprazole is the agonist of DRD2 and 5HT1A receptors but can also antagonize 5HT2A receptor.

The guideline provided by ICH suggested that the study should conduct the further electrocardiographic follow-up if the QTc interval exceeds 10 ms from baseline [21, 22]. The aim of our study was to guide the treatment choice. Therefore, we applied the PRS and three clinical indicators (sex, heart rate and QTc interval at baseline) to distinguish whether patients with schizophrenia have the QTc interval prolongation after 6-week antipsychotic therapy (10 ms as threshold). The ROC analysis showed that the combination of PRS and clinical indicators could significantly distinguish whether patients with schizophrenia have QTc prolongation after antipsychotic treatment in the thresholds 10 ms. Besides, the distinguish ability was more powerful in the first-episode subgroup. It indicated that the combination of PRS and clinical indicators might help clinicians to predict whether the patients with schizophrenia have the QTc interval prolongation after antipsychotic treatment and choose the most appropriate antipsychotic for patients with schizophrenia, especially for the first-episode patients.

There were several limitations in our study. Although our study comprised seven antipsychotics, some antipsychotics which could significantly prolong the QTc interval, such as amisulpride were not taken consideration, genetic effect on more kinds of antipsychotics induced QTc interval prolongation should be analyzed in the future study. Furthermore, although we identified two susceptibility loci associated with antipsychotic-induced QTc interval change in the samples of Han Chinese ancestry, and these identified variants might not be as significant in other ethnic groups. It is necessary to conduct validation studies in other ethnicities and to identify new susceptibility loci for antipsychotic-induced QTc interval change. The result of QTc interval change is still insufficient given the short antipsychotic in the cohort. The QTc interval over a longer treatment period or within a multi-antipsychotic treatment should also be concerned about.

In conclusion, we identified two loci associated with the response to antipsychotic-induced QTc interval change, and the results of ROC analysis indicated that the combination of PRS and clinical data could be applied to predict whether patients with schizophrenia have the side effect of QTc interval prolongation, especially for first-episode patients. Future research should extend these findings to larger samples and different populations to confirm. For the coming study, a larger sample size should be investigated. The extendibility of these findings should be confirmed among different populations.

## Supplementary information

Supplementary Material

## References

[1] Owen MJ, Sawa A, Mortensen PB (2016). Schizophrenia. Lancet.

[2] Lieberman JA (2007). Effectiveness of antipsychotic drugs in patients with chronic schizophrenia: efficacy, safety and cost outcomes of CATIE and other trials. J Clin Psychiatry.

[3] Bozzatello P, Bellino S, Rocca P (2019). Predictive Factors of Treatment Resistance in First Episode of Psychosis: A Systematic Review. Front Psychiatry.

[4] Momen NC, Plana-Ripoll O, Agerbo E, Benros ME, Børglum AD, Christensen MK (2020). Association between Mental Disorders and Subsequent Medical Conditions. N. Engl J Med..

[5] Nachimuthu S, Assar MD, Schussler JM (2012). Drug-induced QT interval prolongation: mechanisms and clinical management. Therapeutic Adv Drug Saf.

[6] Wenzel-Seifert K, Wittmann M, Haen E (2011). QTc prolongation by psychotropic drugs and the risk of Torsade de Pointes. Dtsch Arzteblatt Int.

[7] Huhn M, Nikolakopoulou A, Schneider-Thoma J, Krause M, Samara M, Peter N (2019). Comparative efficacy and tolerability of 32 oral antipsychotics for the acute treatment of adults with multi-episode schizophrenia: a systematic review and network meta-analysis. Lancet (Lond, Engl).

[8] Zai CC, Tiwari AK, Zai GC, Maes MS, Kennedy JL (2018). New findings in pharmacogenetics of schizophrenia. Curr Opin Psychiatry.

[9] Arking DE, Pulit SL, Crotti L, van der Harst P, Munroe PB, Koopmann TT (2014). Genetic association study of QT interval highlights role for calcium signaling pathways in myocardial repolarization. Nat Genet.

[10] Lahrouchi N, Tadros R, Crotti L, Mizusawa Y, Postema PG, Beekman L (2020). Transethnic Genome-Wide Association Study Provides Insights in the Genetic Architecture and Heritability of Long QT Syndrome. Circulation..

[11] Volpi S, Heaton C, Mack K, Hamilton JB, Lannan R, Wolfgang CD (2009). Whole genome association study identifies polymorphisms associated with QT prolongation during iloperidone treatment of schizophrenia. Mol Psychiatry.

[12] Aberg K, Adkins DE, Liu Y, McClay JL, Bukszár J, Jia P (2012). Genome-wide association study of antipsychotic-induced QTc interval prolongation. Pharmacogenomics J.

[13] Yu H, Yan H, Wang L, Li J, Tan L, Deng W (2018). Five novel loci associated with antipsychotic treatment response in patients with schizophrenia: a genome-wide association study. Lancet Psychiatry.

[14] Funk MC, Beach SR, Bostwick JR, Celano C, Hasnain M, Pandurangi A (2020). QTc Prolongation and Psychotropic Medications. Am J Psychiatry.

[15] Purcell S, Neale B, Todd-Brown K, Thomas L, Ferreira MAR, Bender D (2007). PLINK: a tool set for whole-genome association and population-based linkage analyses. Am J Hum Genet.

[16] Leucht S, Samara M, Heres S, Davis JM (2016). Dose Equivalents for Antipsychotic Drugs: The DDD Method. Schizophrenia Bull.

[17] Kanai M, Tanaka T, Okada Y (2016). Empirical estimation of genome-wide significance thresholds based on the 1000 Genomes Project data set. J Hum Genet.

[18] Ramasamy A, Trabzuni D, Guelfi S, Varghese V, Smith C, Walker R (2014). Genetic variability in the regulation of gene expression in ten regions of the human brain. Nat Neurosci.

[19] Zhou Y, Zhou B, Pache L, Chang M, Khodabakhshi AH, Tanaseichuk O (2019). Metascape provides a biologist-oriented resource for the analysis of systems-level datasets. Nat Commun.

[20] Hirokawa M, Morita H, Tajima T, Takahashi A, Ashikawa K, Miya F (2015). A genome-wide association study identifies PLCL2 and AP3D1-DOT1L-SF3A2 as new susceptibility loci for myocardial infarction in Japanese. Eur J Hum Genet: EJHG.

[21] International Conference on Harmonisation; guidance on E14 Clinical Evaluation of QT/QTc Interval Prolongation and Proarrhythmic Potential for Non-Antiarrhythmic Drugs; availability. Notice. Federal Register. 2005; 70:61134–5. 16237860

[22] Hasnain M, Vieweg WVR (2014). QTc interval prolongation and torsade de pointes associated with second-generation antipsychotics and antidepressants: a comprehensive review. CNS Drugs.

[23] West AP, Shadel GS (2017). Mitochondrial DNA in innate immune responses and inflammatory pathology. Nat Rev Immunol.

[24] Kemp JP, Smith PM, Pyle A, Neeve VCM, Tuppen HAL, Schara U (2011). Nuclear factors involved in mitochondrial translation cause a subgroup of combined respiratory chain deficiency. Brain: A J Neurol.

[25] Schon EA, DiMauro S, Hirano M (2012). Human mitochondrial DNA: roles of inherited and somatic mutations. Nat Rev Genet.

[26] Shokolenko I, Venediktova N, Bochkareva A, Wilson GL, Alexeyev MF (2009). Oxidative stress induces degradation of mitochondrial DNA. Nucleic Acids Res.

[27] Yan C, Duanmu X, Zeng L, Liu B, Song Z. Mitochondrial DNA: Distribution, Mutations, and Elimination. Cells. 2019;8:379. 10.3390/cells8040379 PMC6523345 31027297

[28] Kazak L, Reyes A, Holt IJ (2012). Minimizing the damage: repair pathways keep mitochondrial DNA intact. Nat Rev Mol Cell Biol.

[29] Taylor RW, Turnbull DM (2005). Mitochondrial DNA mutations in human disease. Nat Rev Genet.

[30] Lemieux H, Hoppel CL (2009). Mitochondria in the human heart. J Bioenerg Biomembranes.

[31] Wajner M, Amaral AU (2015). Mitochondrial dysfunction in fatty acid oxidation disorders: insights from human and animal studies. Biosci Rep..

[32] Anan R, Nakagawa M, Miyata M, Higuchi I, Nakao S, Suehara M (1995). Cardiac involvement in mitochondrial diseases. A study on 17 patients with documented mitochondrial DNA defects. Circulation.

[33] Lee SR, Han J (2017). Mitochondrial Mutations in Cardiac Disorders. Adv Exp Med Biol.

[34] Desai R, Frazier AE, Durigon R, Patel H, Jones AW, Dalla Rosa I (2017). ATAD3 gene cluster deletions cause cerebellar dysfunction associated with altered mitochondrial DNA and cholesterol metabolism. Brain: A J Neurol.

[35] Gunning AC, Strucinska K, Muñoz Oreja M, Parrish A, Caswell R, Stals KL (2020). Recurrent De Novo NAHR Reciprocal Duplications in the ATAD3 Gene Cluster Cause a Neurogenetic Trait with Perturbed Cholesterol and Mitochondrial Metabolism. Am J Hum Genet.

[36] Baudier J (2018). ATAD3 proteins: brokers of a mitochondria-endoplasmic reticulum connection in mammalian cells. Biol Rev Camb Philos Soc.

[37] Tecalco-Cruz AC, Sosa-Garrocho M, Vázquez-Victorio G, Ortiz-García L, Domínguez-Hüttinger E, Macías-Silva M (2012). Transforming growth factor-β/SMAD Target gene SKIL is negatively regulated by the transcriptional cofactor complex SNON-SMAD4. J Biol Chem.

[38] Khalil H, Kanisicak O, Prasad V, Correll RN, Fu X, Schips T (2017). Fibroblast-specific TGF-β-Smad2/3 signaling underlies cardiac fibrosis. The. J Clin Investig.

[39] Extramiana F, Milleron O, Elbitar S, Uccellini A, Langeois M, Spentchian M (2018). High prevalence of ventricular repolarization abnormalities in people carrying TGFβR2 mutations. Sci Rep.

[40] Ferreira RR, Abreu R, da S, Vilar-Pereira G, Degrave W, Meuser-Batista M (2019). TGF-β inhibitor therapy decreases fibrosis and stimulates cardiac improvement in a pre-clinical study of chronic Chagas’ heart disease. PLoS Neglected Tropical Dis.

[41] Kim JO, Park JH, Kim T, Hong SE, Lee JY, Nho KJ (2018). A novel system-level approach using RNA-sequencing data identifies miR-30-5p and miR-142a-5p as key regulators of apoptosis in myocardial infarction. Sci Rep..

[42] Kishore R, Verma SK, Mackie AR, Vaughan EE (2013). Abramova T v, Aiko I, et al. Bone marrow progenitor cell therapy-mediated paracrine regulation of cardiac miRNA-155 modulates fibrotic response in diabetic hearts. PloS One.

[43] Xu Y, Yue W, Yao Shugart Y, Li S, Cai L, Li Q (2016). Exploring Transcription Factors-microRNAs Co-regulation Networks in Schizophrenia. Schizophrenia Bull.

[44] Yohn SE, Foster DJ, Covey DP, Moehle MS, Galbraith J, Garcia-Barrantes PM (2020). Activation of the mGlu(1) metabotropic glutamate receptor has antipsychotic-like effects and is required for efficacy of M(4) muscarinic receptor allosteric modulators. Mol Psychiatry.

[45] Maas SCE, Mens MMJ, Kühnel B, van Meurs JBJ, Uitterlinden AG, Peters A (2020). Smoking-related changes in DNA methylation and gene expression are associated with cardio-metabolic traits. Clin Epigenetics.

[46] Yamauchi T, Kang G, Hiroi N (2021). Heterozygosity of murine Crkl does not recapitulate behavioral dimensions of human 22q11.2 hemizygosity. Genes, Brain, Behav.

[47] Chen X, Barajas-Martínez H, Xia H, Zhang Z, Chen G, Yang B (2021). Clinical and Functional Genetic Characterization of the Role of Cardiac Calcium Channel Variants in the Early Repolarization Syndrome. Front Cardiovascular Med.

[48] Moody CL, Funk AJ, Devine E, Devore Homan RC, Boison D, McCullumsmith RE (2020). Adenosine Kinase Expression in the Frontal Cortex in Schizophrenia. Schizophrenia Bull.

[49] Park T-J, Boyd K, Curran T (2006). Cardiovascular and craniofacial defects in Crk-null mice. Mol Cell Biol.

[50] Liang D, Xue Z, Xue J, Xie D, Xiong K, Zhou H (2021). Sinoatrial node pacemaker cells share dominant biological properties with glutamatergic neurons. Protein Cell.

[51] Yokotsuka-Ishida S, Nakamura M, Tomiyasu Y, Nagai M, Kato Y, Tomiyasu A (2021). Positional cloning and comprehensive mutation analysis identified a novel KDM2B mutation in a Japanese family with minor malformations, intellectual disability, and schizophrenia. J Hum Genet.

[52] Schizophrenia Working Group of the Psychiatric Genomics Consortium. Biological insights from 108 schizophrenia-associated genetic loci. Nature. 2014;511:421–7. 10.1038/nature13595 PMC4112379 25056061

