# Supplementary material for: ATAD3B and SKIL polymorphisms associated with antipsychotic-induced QTc interval change in patients with schizophrenia: a genome-wide association study
Source: Transl Psychiatry. 2022 Feb 8;12:56. doi: 10.1038/s41398-022-01825-0 (PMC8825824; doi:10.1038/s41398-022-01825-0)
Supplement: Supplementary file 1 — Supplementary Material [file 41398_2022_1825_MOESM1_ESM.doc]

**Supplementary** **Material**

**Supplementary Methods**

**1. Subject enrollment**

The cohort was from the Chinese Antipsychotics Pharmacogenomics Consortium (including five research centers: Peking University Sixth Hospital, West China Hospital of Sichuan University, the Second Xiangya Hospital of Central South University, Beijing Anding Hospital Affiliated to Capital Medical University, and Beijing HuiLongGuan Hospital; the Consortium leads 32 psychiatric hospitals in China in total).

This study was conducted in accordance with the Declaration of Helsinki. The protocol was approved by the institutional ethics review boards at each site, and written informed consent was obtained.

Inclusion criteria: (1) Diagnosed with schizophrenia based on the Structured Clinical Interview of the Diagnostic and Statistical Manual of Mental Disorders, fourth edition (DSM-IV); (2) Aged from 18 to 45 years; (3) Han Chinese ancestry; (4) Total scores of the Positive and Negative Syndrome Scale (PANSS) were more than 60, and three positive items scored more than four at least; (5) Physically healthy with all laboratory parameters within normal limits; (6) Could be treated with oral medication; (7) Provide informed consent.

Exclusion criteria: (1) Diagnosed with other mental disorders met the criteria of DSM-IV; (2) With unstable physical diseases, malignant syndrome or acute dystonia, well documented histories of epilepsy and hyperpyretic convulsion; (3) Required long-acting injectable antipsychotics; (4) Regularly toke with clozapine during the past month; (5) Treated with electroconvulsive therapy during the last month; (6) Had previously attempted suicide, or had experienced the symptoms of severe excitement and agitation; (7) Abnormal liver or renal function; (8) without legal guardian; (9) Had QTc prolongation, a history of congenital QTc prolongation, or myocardial infarction within the past 6 months; (10) Pregnant or breastfeeding; (11) Had a contraindication to any of the drugs to which they could be assigned.

Eligible patients were randomly assigned to seven groups and received 6-week antipsychotic treatment. (Detailed randomization and procedures were described in the appendix).

**2. Randomization and masking**

Eligible patients were randomly assigned (1:1:1:1:1:1) to six groups (aripiprazole, olanzapine, quetiapine, risperidone, ziprasidone, or one of the first-generation antipsychotics [haloperidol or perphenazine]). Those randomly assigned to the first-generation antipsychotics group were subsequently randomly assigned (1:1) to haloperidol or perphenazine. Group assignment was established with a Microsoft Excel randomization generator without any stratification factors. The random allocation sequence was generated by a trained research assistant who had no further role in the trial, and was concealed until after baseline assessments. The researchers doing both the baseline and the follow-up assessments were masked to the group assignments of each participant. Patients and psychiatrists were unmasked to assigned antipsychotics.

**3. Clinical Procedure**

All patients were given a screening questionnaire, which recorded the demographic and clinical information. We did baseline assessments to ensure that participants met inclusion criteria. Patients who were already taking antipsychotic medications were obliged to switch to their newly assigned drug within 1 week of randomization. Within 2 weeks of randomization, psychiatrists from the study adjusted the dosages on the basis of treatment effectiveness, in keeping with the study protocol (olanzapine doses could range from 5 mg to 20 mg per day, risperidone from 2 mg to 6 mg per day, quetiapine from 400 mg to 750 mg per day, aripiprazole from 10 mg to 30 mg per day, ziprasidone from 80 mg to 160 mg per day, haloperidol from 6 mg to 20 mg per day, and perphenazine from 20 mg to 60 mg per day). The dosage of antipsychotics then remained unchanged throughout the study. If the psychiatrists decided that a patient’s response was not adequate or the patient decided to drop out of the study, treatment was discontinued and the last observation was carried forward to represent treatment response. Patients with adequate responses continued treatment until the end of the study.

**4. Genotyping**

Genomic DNA was extracted with the QIAamp DNA Mini Kit (QIAGEN, Hilden, Germany). The samples were genotyped with Illumina Human Omni ZhongHua-8 Beadchips (Illumina, San Diego, CA, USA), which were designed for Chinese populations. Quality control was done before the association analysis. Samples were excluded if the genotype call rate was less than 98%, in the case of gender discordance, if they were first-degree or second-degree relatives, or if they were genetic outliers. SNPs were excluded if minor allele frequency was less than 0.01, the genotype call rate was less than 98%, or p values for Hardy-Weinberg equilibrium

were less than 1 × 10^-5^. Genotype imputation for the sample was done with the pre-phasing imputation stepwise approach implemented in IMPUTE2 and SHAPEIT (Version 2.r727). Haplotypes derived from phase I of the 1000 Genomes Project (release version 3) were used as references. SNPs with imputation quality scores below a set threshold (info score <0.9) were excluded from further analyses. All genomic locations are given as National Center for Biotechnology Information Build 37 coordinates.

**Supplementary Figures**

**Supplementary Figure 1: Quantile–quantile (A) and Manhattan plots (B)**

(A) The plot was generated with principal-component-analysis-adjusted data. λ _GC_ =1.004

(B) Genome-wide p values were plotted against their respective chromosomal positions. The blue line represents the significance level for follow-up (1× 10^-5^), the red line represents the genome-wide significance level (5× 10^-8^).


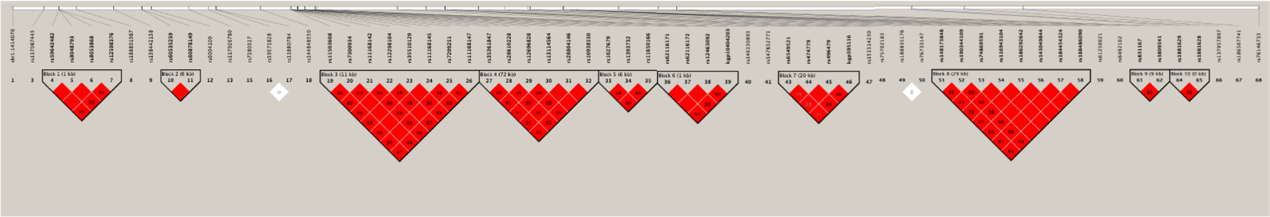


**Supplementary Figure 2. LD block plot of 68 suggestive SNPs.**

**Supplementary Figure 3: Regional association plots for chromosomes 1p36.33 and 3q26.2, showing genome-wide significant SNPs rs200050752 (A) and rs186507741 (B)**

For genotyped and imputed SNPs passing quality-control measures in the genome-wide association study, –log10 (p) was plotted as a function of genomic position. All other SNPs are color coded according to the strength of linkage disequilibrium (as measured by r²) with this index SNP. Linkage disequilibrium values were established on the basis of the 1000 Genomes Project Asian data (November, 2014). Estimated recombination rates with samples from the 1000 Genomes Project are represented by blue lines, and genes within the regions of interest annotated from the University of California, Santa Cruz Genome Browser are shown as arrows. SNP, single-nucleotide polymorphism.

**Supplementary Figure 4. Gene expression pattern of the two associated genes in human tissues of artery, heart and brain in GTEX database.**

The red plot refers to the artery tissues, the yellow plot refers to the brain tissues, and the purple plot refers to the heart tissues. *SKIL* (A) *and ATAD3B* (B) were preferentially expressed in human artery tissues (red plot), and the SKIL was preferentially expressed in atrial appendage, while the ATAD3B was preferentially expressed in cerebellum and cerebellum hemisphere.

**Supplementary Figure 5. Polygenic risk scores**

(A) Bar plot from PRSice showing results at broad *P*-value thresholds for atrial fibrillation PRS predicting QTc interval change. A bar for the best-fit PRS from the high-resolution run is also included.

(B) High-resolution PRSice plot for SCZ predicting MDD status. The thick line connects points at the broad *P*-value thresholds of (A)

**Supplementary Figure 6. Path analysis**

The sex, heart rate and QTc interval at baseline have significantly direct effect on antipsychotic-induced QTc interval change. There were indirect effect between sex and baseline QTc interval, as well as baseline QTc interval and baseline heart rate.


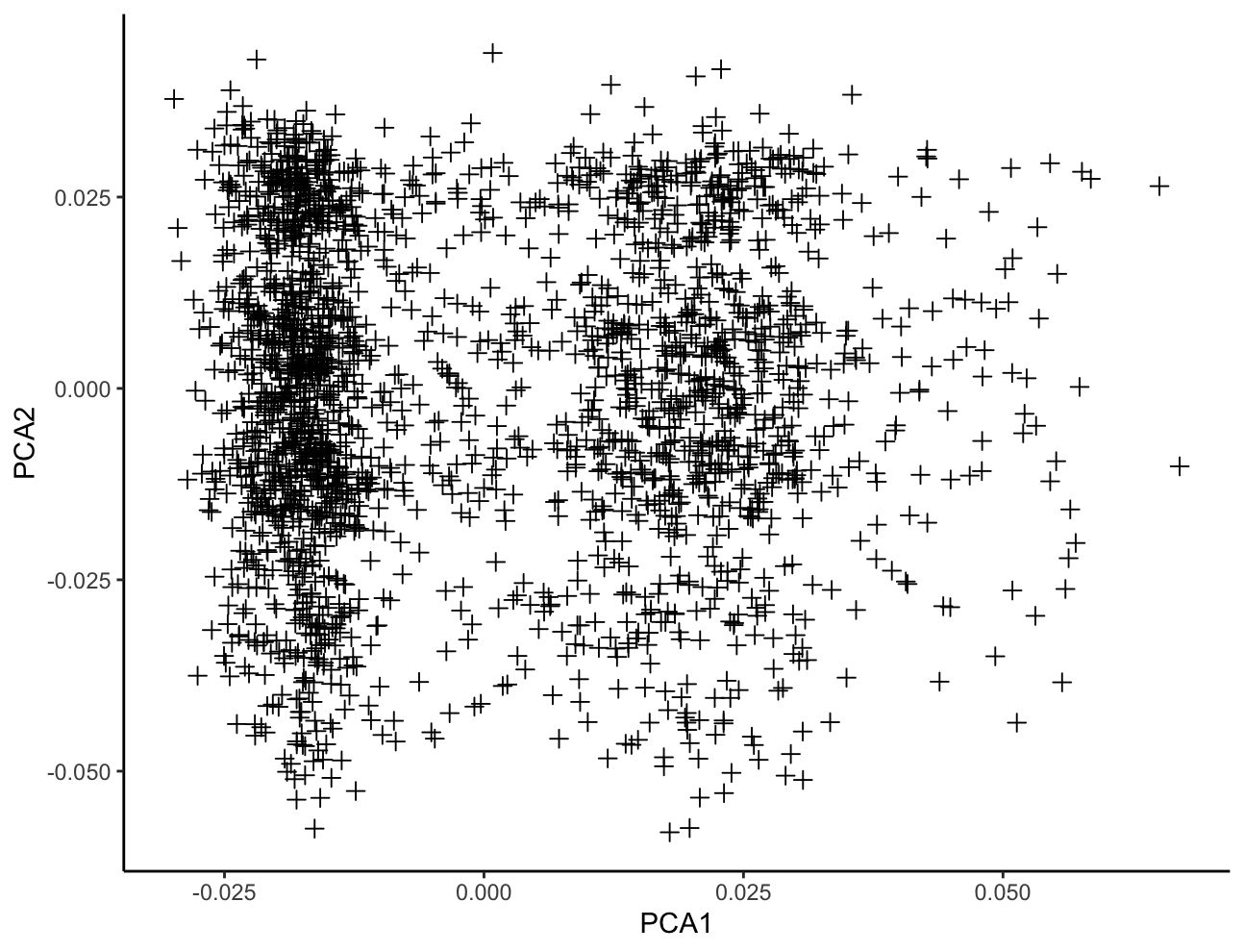


**Supplementary Figure 7. PCA plot of population stratification**

**Supplementary Tables**

**Supplementary Table 1. Pathways and disease enrichment.**

| GO | Description | Log10(*P*) |
| --- | --- | --- |
| Pathways |  |  |
| GO:0010720 | Positive regulation of cell development | -2.97 |
| GO:0007283 | Spermatogenesis | -2.19 |
| Disease* |  |  |
| C0201976 | Creatinine measurement, serum (procedure) | -4.00 |
| [C0017654](http://www.disgenet.org/browser/0/0/3/0/diseaseid__C0017654-source__ALL/_b./) | Glomerular filtration rate | -3.80 |
| [C0278996](http://www.disgenet.org/browser/0/0/3/0/diseaseid__C0278996-source__ALL/_b./) | Malignant head and neck neoplasm | -2.70 |
| [C3887461](http://www.disgenet.org/browser/0/0/3/0/diseaseid__C3887461-source__ALL/_b./) | Head and neck carcinoma | -2.70 |
| [C0018790](http://www.disgenet.org/browser/0/0/3/0/diseaseid__C0018790-source__ALL/_b./) | Cardiac arrest | -2.50 |
| [C1837461](http://www.disgenet.org/browser/0/0/3/0/diseaseid__C1837461-source__ALL/_b./) | Scoliosis, isolated, susceptibility, 3 | -2.10 |
| [C0525045](http://www.disgenet.org/browser/0/0/3/0/diseaseid__C0525045-source__ALL/_b./) | Mood disorders | -2.10 |

*, the disease enrichment was based on the DisGeNET dataset.

**Supplementary Table 2. Antipsychotic-specific genome-wide association results of QTc interval change.**

|  | CHR | BP | SNP | A1 | Nearby gene | Beta | SE | P |
| --- | --- | --- | --- | --- | --- | --- | --- | --- |
| Risperidone | 6 | rs143045719 | 146347539 | C | GRM1 | 117.6 | 6.605 | 1.66E-10 |
|  | 12 | rs117241889 | 95912601 | T | USP44 | 47.28 | 5.653 | 3.44E-08 |
|  | 12 | rs144273052 | 95938956 | C | USP44 | 49.99 | 5.625 | 4.13E-08 |
| Olanzapine | 12 | rs141604954 | 121970208 | T | KDM2B | 101.7 | 6.202 | 1.68E-09 |
|  | 17 | rs140577770 | 71757268 | T | LINC00469 | 119.7 | 6.061 | 3.77E-09 |
|  | 8 | rs151101057 | 25861413 | A | EBF2 | 72.17 | 5.771 | 1.85E-08 |
| Quetiapine | 10 | rs149596073 | 76265363 | T | ADK | -123.9 | -6.096 | 3.22E-09 |
|  | 12 | rs192721831 | 80643818 | A | OTOGL | -123.8 | -6.075 | 3.70E-09 |
| Aripiprazole | 12 | rs149121493 | 2417373 | C | CACNA1C | 64.86 | 6.233 | 1.66E-09 |
|  | 7 | rs149011939 | 43942925 | C | URGCP | 130.6 | 6.084 | 3.43E-09 |
|  | 12 | rs11168673 | 49057823 | T | SNORA2B | 92.77 | 6.082 | 3.46E-09 |
|  | 12 | rs11168675 | 49062652 | C | KANSL2 | 92.77 | 6.082 | 3.46E-09 |
|  | 7 | rs143034580 | 43844859 | A | BLVRA | 130.5 | 6.074 | 3.63E-09 |
|  | 12 | rs142212460 | 63273306 | G | PPM1H | 103.4 | 5.997 | 5.66E-09 |
|  | 12 | rs141713937 | 2373211 | T | CACNA1C | 55.72 | 5.938 | 8.38E-09 |
|  | 1 | rs141265578 | 58371768 | A | DAB1 | 127.7 | 5.922 | 8.64E-09 |
|  | 14 | rs79797842 | 94436739 | T | ASB2 | 100.2 | 5.813 | 1.75E-08 |
|  | 3 | rs145379527 | 20147341 | G | KAT2B | 68.48 | 5.598 | 4.85E-08 |
| Ziprasidone | 17 | rs187008435 | 1354343 | A | CRK | 129.3 | 6.234 | 1.54E-09 |
|  | 2 | rs144350515 | 242824616 | G | LINC01237 | 76.74 | 6.209 | 1.65E-09 |
|  | 18 | rs190925085 | 40078639 | C | LINC00907 | 131.4 | 6.084 | 3.46E-09 |
|  | 8 | rs144541917 | 85752719 | A | RALYL | 89.1 | 5.831 | 1.48E-08 |
|  | 4 | rs28989203 | 144275543 | T | GAB1 | 52.07 | 5.631 | 4.04E-08 |

CHR, Chromosome; SNP, single nucleotide polymorphism.
